# Muscle Damage Biomarkers in Congestion Weeks in English Premier League Soccer Players: A Prospective Study for Two Consecutive Seasons

**DOI:** 10.3390/ijerph18157960

**Published:** 2021-07-28

**Authors:** Álvaro García-Romero-Pérez, Francisco Javier Ordonez, Fernando Reyes-Gil, Elena Sonsoles Rodríguez-López, Ángel Oliva-Pascual-Vaca

**Affiliations:** 1 Injury Prevention and Rehabilitation Department, Watford FC, Watford WD18 0ER, UK; agromero@ucjc.edu; 2 Physiotherapy Department, Camilo José Cela University, 28692 Madrid, Spain; freyes@ucjc.edu; 3 Physiotherapy Department, Universidad of Sevilla, 41004 Sevilla, Spain; angeloliva@us.es; 4 School of Sports Medicine, University of Cádiz, 11003 Cádiz, Spain; franciscojavier.ordonez@uca.es; 5 Escuela de Osteopatía de Madrid, 28002 Madrid, Spain

**Keywords:** soccer, creatine kinase, muscle damage, fatigue, GPS, high speed running

## Abstract

The current study was conducted to compare muscle damage biomarkers in single- vs. multi-match weeks in elite soccer players for two consecutive seasons. A secondary objective was to analyze the influence of playing position and exposure time on muscle damage in single- vs. multi-match weeks. This is a prospective cohort study performed in a professional elite soccer club in the English Premier League during the 2018–2019 and 2019–2020 seasons up until the lockdown due to the COVID-19 pandemic. Data were collected in the Medical Department Room of an English Premier League Club before and after the soccer game from a total of 29 elite soccer players (mean ± S.D.; age = 27.59 ± 3.83 years; height = 1.83 ± 0.05 m; body mass = 80.16 ± 7.45 kg) who were enrolled in the club during both seasons. The main outcome measurements were creatine kinase (CK), weight, lean mass, % fat DEXA, high speed running, total distance, density of total distance and high-speed running and wellbeing questionnaires. Significance was set at *p* < 0.05. Players who completed more than 60 min in the previous game had significantly increased pregame CK levels and fatigue in multi-match weeks. Midfielders had both significantly increased pregame CK and muscle soreness in multi-match weeks. Midfielders and players with an exposure time of at least 60 min showed higher pregame CK values that should play a key role for deciding substitutions.

## 1. Introduction

Professional sport has been affected by the COVID-19 pandemic [1,2]. Since lockdown, top soccer leagues in Europe have faced a congested schedule with multiple matches per week and short recovery periods in order to complete the most recent season. Some evidence has started to show that the congested weeks after the COVID-19 lockdown increased the number of injuries, with the injury rate per game being 0.84 after lockdown, compared to 0.27 prior to the COVID-19 pandemic [2].

This extraordinary situation may have several consequences in clinical practice, provided that insufficient recovery may reduce athletic performance and increase the risk of sport-related injuries [2,3]. In fact, it is widely accepted that kinetic models predict higher overall injury burden for successful teams competing in both national and European club competitions [4]. It is important to take into account that even a recovery period of 72 h post-match may not be long enough to completely restore the homeostatic balance in soccer players [5,6].

In a more detailed way, creatine kinase (CK) levels have been used to monitor muscle damage in elite soccer players [7,8,9,10] and other sports [11,12]. A recent systematic review pointed out that a real match format induced greater levels of CK compared to simulation protocols [5]. Most studies conducted in real-world scenarios were single-match experiments [13] or short-term prospective studies [7,14,15,16]. Much less information is available on long-term prospective studies in top soccer leagues [16]. To the best of our knowledge, no study has compared muscle damage in single- and multi-match weeks during the entire season in an elite professional soccer environment. In addition, the potential effects of playing different positions have not traditionally been accounted for by previous authors [7,17], despite the fact that there are different position-specific physical demands in professional football [18].

The current prospective cohort study was conducted to compare muscle damage in single- vs. multi-match weeks in Premier League soccer players for two seasons in a row. A secondary objective was to analyze the influence of playing position on muscle damage in congested versus standard schedules.

## 2. Materials and Methods

### 2.1. Design

This is a prospective cohort study performed in a professional elite soccer club in the English Premier League during the 2018–2019 and 2019–2020 seasons up until the lockdown due to the COVID-19 pandemic. The current study was approved by the Ethics Committee of Camilo José Cela University (Madrid, Spain) and adhered to the tenets of the Declaration of Helsinki [19]. All participants gave their consent to participate after having been informed of the study’s objectives and procedures. All data were anonymous and confidential in line with new European data protection laws [20].

### 2.2. Study Participants

Data were collected from 29 elite soccer players (mean ± S.D.; age = 27.6 ± 3.8 years; height = 1.83 ± 0.05 m; body mass = 80.16 ± 7.45 kg) who were enrolled in the club during both seasons. Goalkeepers were excluded due to their specific role in the team. The positional breakdown assigned to the players was: defender, midfielder, and striker [13,21,22]. As exclusion criteria, samples of blood were not taken from the player if they had any injury or had any muscle knock during the game prior to the test. In multigame weeks, data of the player were not included if the player had not played the previous game.

### 2.3. Procedures

Data were related to 38 matches in the 2018–2019 season and 29 matches in the 2019–2020 season. Since fixtures were not distributed homogeneously during the whole season, weeks were considered as multi-match weeks or single-match weeks when the time between matches was shorter or longer than 4 days (96 h), respectively [23]. So, for the games that had less than 4 days between them, samples were taken pre- and post-match, and these samples were designated as being from a multi-match week. Otherwise, for those games that had more than 4 days between them, the samples were designated as being from a single-match week, and the samples were taken with the same procedure.

Match performance data were collected across both seasons by the English Premier League—with TRACAB (Chyroego Corporation, New York, NY, USA) in 2018–2019 and Second Spectrum (Los Angeles, CA, USA) in 2019–2020 installed at the stadiums of the home team. The data supplied provided different information about total distance and different velocities of the players during the game. Kick off time was from 12:30 to 19:00 depending on the fixture and television broadcasting. To develop the player’s activity profiles, movements were coded into the following categories and speed thresholds: standing (0–0.2 m.s^−1^), walking (0.2–2 m.s^−1^), jogging (2–4 m.s^−1^), running (4–5.5 m.s^−1^), high-speed running (5.5–7 m.s^−1^) and sprinting (>7 m.s^−1^), as in previous investigations [24,25]. The density of total distance and high-speed running, dividing total distance and high speed running meters by the number of minutes played, was also calculated.

The primary outcome measure was muscle damage through CK analysis. Blood test samples were collected in two moments. Pre-game and post-game CK were taken the days prior to and after the game, between 09:00 and 10:00 without having breakfast, when the players arrived at the training ground facilities. The samples were collected in a standing position by the same member of the club staff, using a sterile lancet (Roche, Mannheim, Germany) in combination with a spring-loaded AccuChek lancet device (Roche, Mannheim, Germany). A 30 μL capillary blood sample was placed on the measurement strip and analyzed by Reflotron Plus (Reflotron Systems, Roche, Mannheim, Germany) system according to the manufacturer’s recommendations. Capillary blood was analyzed using this method and displayed an intraassay reliability of <3% coefficient of variation [26]. Pre-game training consists of a very light session with some warming up drills and mobility exercises.

Wellbeing questionnaires were collected everyday through a software program (Soccer System Pro, Barcelona, Spain) on an iPad Air (Apple, Cupertino, CA, USA) located in the changing room of the players. Players were familiarized with this questionnaire, which was distributed according to previous recommendations [27,28,29] and comprised different questions such as fatigue, muscle soreness, sleep quality and stress scored on a five-point Likert scale (values 1–5 with 1 point of increment, where 1 means very good ratings and 5 very bad). The number of sleep hours was also collected. Although these scores were collected every day, for this analysis, only data related to the day prior to the game were considered.

Body fat and lean mass percentages were evaluated by means of dual-energy x-ray absorptiometry (DEXA) using a total body scanner (QDR Explorer W, Hologic, MA, USA; fan-bean technology, software for Windows XP version 12.6.1) according to the manufacturer’s procedure. All the scanning and analyses were performed by the same specialist to ensure consistency.

### 2.4. Statistical Analysis

All statistical tests were performed using the package IBM SPSS Statistics v. 26.0 (SPSS Science, Chicago, IL, USA). A descriptive study was carried out to analyze the data. Continuous variables were presented as mean values and standard deviation (SD) along with 95% confidence intervals (CI). When appropriate, data were provided as percentages. The Shapiro–Wilk test was used to identify normal distribution of the data. The Mann–Whitney U test was used to analyze differences between multi-match weeks and single-match weeks. One-way ANOVA or multivariate data analysis was used to test the profile of the values, depending on the playing position, in the congested weeks and non-congested weeks. Analysis was performed at the 95% confidence level. Significance was set at *p* < 0.05.

## 3. Results

Out of the 55 analyzed matches, 22 were considered to belong to multi-match weeks and 33 to single-match weeks. In addition, CK pre-match multi-match data involved a mean of 9.77 players per match, while single-match data involved a mean of 9.75 players per match.

Table 1 shows a comparative assessment of muscle damage, wellbeing, body composition and match performance data in single- vs. multi-match weeks in professional soccer players playing in the English Premier League. In general terms, significant differences were found for GPS total distance and density total distance, with multigame weeks showing higher values than single-match weeks. No other significant differences were found for CK values or any other variable.

**Figure 1:**
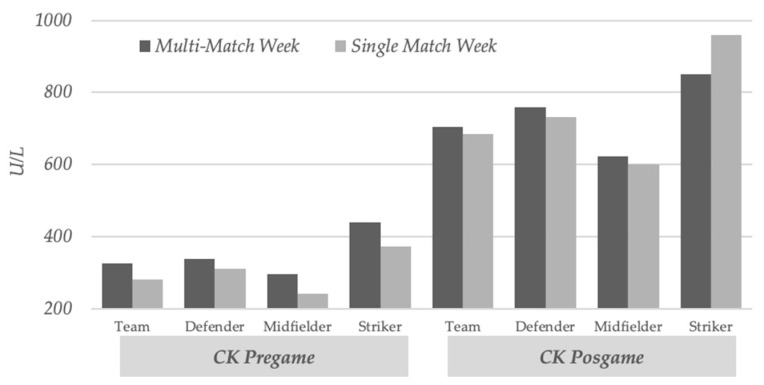
Muscle damage through creatine kinase analysis in multi-match vs. single-match weeks in soccer players that completed more than 60 min in the game and according to player position.

**Figure 2:**
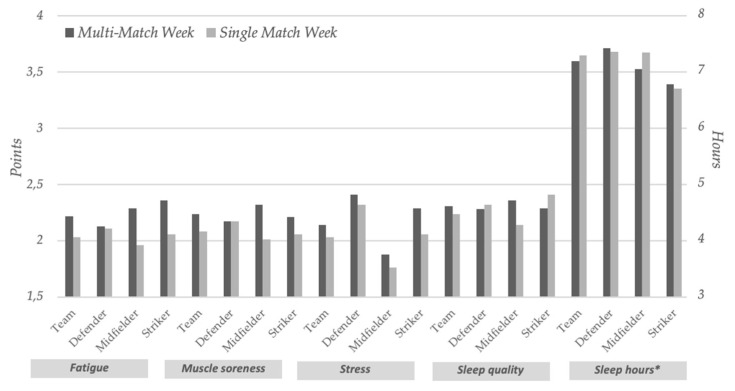
Wellbeing questionnaire data in multi-match vs. single-match weeks in soccer players that completed more than 60 min in the game and according to player position. (*) The sleep hours are represented on the right axis.

**Figure 3:**
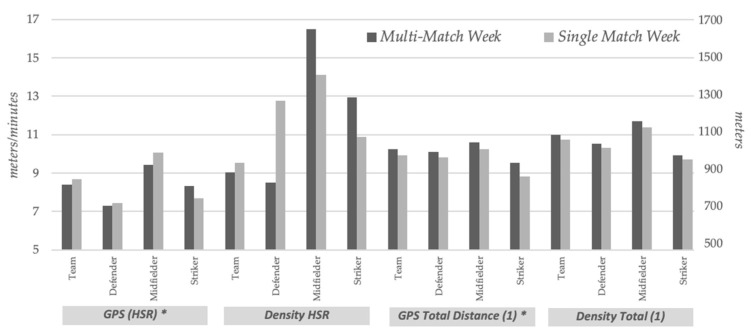
Match performance data in multi-match vs. single-match weeks in soccer players that completed more than 60 min in the game and according to player position. (1) For data representation, GPS total distance mean and density total mean were divided by 10. (*) GPS and GPS total distance are represented on the right axis.

Players who completed more than 60 min in the previous game had significantly increased pregame CK levels in multi-match compared to single-match weeks (324.97 ± 191.40 vs. 279.90 ± 158.3; *p* = 0.029) (Figure 1). In addition, these players also had significantly higher levels of fatigue (2.22 ± 0.80 vs. 2.03 ± 0.69; *p* = 0.015) (Figure 2) and density total distance (109.97 ± 10.30 vs. 107.50 ± 11.53; *p* = 0.036) (Figure 3) in multi-match vs. single-match weeks. The most striking finding among players who played less than 60 min was a postgame CK that was significantly increased in multi-match vs. single-match weeks (556.65 ± 291.10 vs. 384.47 ± 163,87; *p* = 0.029). No more significant changes were found in this group. These results are listed in Table 2.

Regarding player position, our results clearly demonstrate that midfielders had pregame CK values that were significantly increased in multi-match weeks when compared to single-match weeks (295.16 ± 185.26 vs. 240.58 ± 134.97; *p* = 0.045). Similarly, midfielders’ muscle soreness (2.36 ± 0.81 vs. 2.14 ± 0.82; *p* = 0.047) and total distance (10,592.38 ± 10,592.38 vs. 10,233.59 ± 1482.91; *p* = 0.033) were also significantly higher in multi-match vs. single-match weeks. Conversely, no significant changes were found in defenders and strikers. These results are summarized in Table 3.

## 4. Discussion

To the best of our knowledge, this was the first study that compared muscle damage in single- vs. multi-match weeks for two consecutive seasons in English Premier League soccer players.

Our results clearly demonstrate that pre-game levels of CK were significantly higher in congested compared to non-congested weeks when players played more than 60 min. Similarly, internal training load expressed as fatigue and muscle soreness were also significantly increased. As the biomarker is an individual independent value, clinically this difference suggests that changes in pre-game CK levels intra-individually in a congested week would help us to understand how close the player is to the baseline of a single-match week. This pre-game CK level could be taken as an important factor by the coaches and the medical department when deciding the availability of players for the next game. Conversely, in spite of post-game CK levels being higher in multi-match weeks, differences were not statistically significant with respect to single-match weeks. The latter finding could be explained considering that in periods of congested scheduling, soccer players reduced the number of low and medium intensity actions they participated in, but maintained the number of higher intensity actions [30]. In addition, Carling et al. (2012) [17] reported that injury risk was generally unaffected during a prolonged period of fixture congestion (8 consecutive official matches in 26 days), provided that squad rotation and post-match recovery strategies were conducted in an appropriate way. Other investigations have shown that injury risk is affected by congested weeks, including in an 11-year follow up Champions League injury study [31].

Our study also shows that there is no difference in total distance between players due to playing in a single- or multi-match week, which is similar to other previous research [32], and postgame CK levels were similar to those reported by Bok and Jukic (2020) in Croatian national-team players. In both studies, post-game CK levels were determined in the morning of the first day after the match, less than 24 h post-match. Higher CK values have been reported in previous studies at 24–36 h [33], 48 h [34] and 72 h [35] post-match in professional soccer players. This fact could explain, at least in part, the higher pre-game CK levels in congested weeks observed in the current cohort study when recovery periods between consecutive matches were <4 days. In addition, it should be pointed out that all latter studies were conducted in competitive environments, meaning that recovery strategies were expected to be applied [34,35]. This idea will match with other authors that conclude that more than four days is needed for the complete recovery of the biomarkers to the pregame level [5].

It is widely accepted that there are position-specific differences in soccer players’ match performance [16,36,37]. As was hypothesized, player position may also have a significant impact on muscle damage, in spite of most previous authors not accounting for it [7,17,33,38]. The current results also demonstrate that midfielders had higher pregame CK levels when compared to defenders and forwards. In this respect, it was also found that midfielders performed a significantly increased density of high-intensity work. Similar results were previously reported by Souglis et al. (2018) in a short-term prospective study on professional soccer players from Greece’s major league. In addition, Bok and Jukic (2019) recently reported that muscle damage was higher in Croatian national-team players with better aerobic capacity because they could exert a greater number of high-intensity bouts and rapid eccentric contractions during a soccer match. For the reasons already mentioned, positional analysis could be of practical use to coaches and practitioners in order to emphasize the necessity of individualized training and recovery protocols.

Recent studies have emphasized that professional soccer matches can be carried out safely during the COVID-19 pandemic [39]. It was shown that in the Bundesliga, German football’s first division, players were more likely to have injuries after lockdown than before. According to our results, player substitution and rotation strategies, as other studies have shown [3], with special emphasis on midfielders, should be strongly encouraged in periods of fixture congestion. Therefore, the five-substitute rule, a temporary amendment to the Laws of the Game approved by the International Football Association Board (IFAB) for the end of the most recent season, facilitates the decisions about substitutions in order to improve performance and diminish the probability of injury [40].

The strengths of the current prospective cohort study include that it was conducted in a professional soccer club in the English Premier League for two consecutive seasons. In contrast to previous cross-sectional studies [6] or short-term prospective studies, we have assessed a total of 22 multi-match and 33 single-match weeks. Secondly, considering the fact that CK responses in soccer players are individualized (Silva et al. 2014), it is noteworthy to point out that it involved the same players for both seasons, although it reduced the sample size because new players were not included in data from the second season. Thirdly, checking postgame CK levels early in the morning of the following match day, rather than in a strict 24 h cycle post-match, saved time in the design and applied individualized training loads and recovery protocols according to their own figures.

### Study Limitations

The present study had some limitations that should be also addressed. Firstly, data from a single club were assessed, meaning that the current results may have limited application. Secondly, considering the fact that collecting data during official top-level competitions is a complex institutional mission within a particular ecological environment, we could not repeat postgame CK levels in a strict 24- or 48-h cycle after the end of the match, as shown in previous studies on this topic [33,34].

Thirdly, it is well-known in the related literature that CK values are individualized and highly dependent on multiple factors as lean mass [41], race [42], the level of the athlete [43] and position. Standardized CK levels cannot be a value reference [9,10], but an individualized profile of CK levels at the same time of the week (day prior to the game) could be a good internal load reference for exploring how the muscle is returning to normal individualized levels.

## 5. Conclusions

It was concluded that pregame CK levels were significantly increased in multi-match weeks when players had less than 4 days of recovery between consecutive matches. Conversely, no significant changes were found in postgame CK levels, suggesting that player rotation and early recovery strategies were adequate in both single- and multi-match weeks. Regarding playing position, midfielders exhibited higher pregame CK levels when compared to forwards and defenders. Similar results were reported in soccer players who played more than 60 min in the previous game. Accordingly, individualized training loads and recovery protocols are strongly encouraged. Furthermore, prospective cohort studies, involving a larger sample of clubs from elite soccer leagues, are necessary to confirm the current findings.

## Figures and Tables

**Table 1:** Comparative assessment of muscle damage, wellbeing, body composition and match performance data in multi-match vs. single-match weeks in professional soccer players playing in the English Premier League.

	Multi-Match Week			Single-Match Week			
	95% CI			95% CI			
	Mean [SD]	Lower	Upper	Mean [SD]	Lower	Upper	*p* ^†^
CK PREGAME (U/L)	329.83 [199.58]	303.00	356.66	313.04 [176.55]	293.69	332.40	0.636
CK POSTGAME (U/L)	663.25 [375.62]	598.82	727.68	658.76 [319.44]	610.39	707.12	0.539
WEIGHT (kg)	80.06 [7.47]	79.14	80.98	80.09 [7.47]	79.35	80.83	0.991
LEAN MASS (kg)	69.63 [67.76]	68.87	70.39	69.76 [66.94]	69.17	70.36	0.755
% FAT DEXA	13.41 [1.51]	13.24	13.58	13.33 [1.53]	13.19	13.46	0.373
FATIGUE ^‡^	2.12 [0.83]	2.02	2.22	2 [0.73]	1.93	2.07	0.073
MUSCLE SORENESS ^‡^	2.15 [0.81]	2.05	2.25	2.03 [0.7]	1.96	2.10	0.067
STRESS ^‡^	2.15 [1.11]	2.01	2.28	2.01 [1.04]	1.91	2.11	0.125
SLEEP QUALITY ^‡^	2.16 [0.75]	2.07	2.26	2.15 [0.79]	2.07	2.22	0.574
SLEEP HOURS	7.28 [1.02]	7.15	7.40	7.39 [0.99]	7.29	7.49	0.207
GPS (HSR) (m)	716.43 [350.86]	672.55	760.31	724.39 [436.75]	681.02	767.76	0.451
DENSITY HSR (m/min)	10.12 [5.78]	9.39	10.84	10.31 [5.52]	9.76	10.86	0.607
GPS TOTAL DISTANCE (m)	8544.52 [3431.35]	8115.36	8973.68	8154.28 [3323.48]	7824.26	8484.30	0.041 *
DTD (m/min)	110.89 [11.61]	109.44	112.35	108.91 [13.8]	107.54	110.29	0.038 *

CK, creatine kinase; kg, kilograms; GPS, global positioning system; HSR, high-speed running; DTD, density total dis-tance; m, meters; min, minutes; SD, standard deviation; CI, confidence interval. *p* ^†^ value: based on U Mann–Whitney test; ^‡^ Wellbeing questionnaire, scored on a five-point Likert scale (values 1–5: 1—very good and 5—very bad); * *p* value < 0.05.

**Table 2:** Comparative assessment of muscle damage, wellbeing, body composition and match performance data in multi-match vs. single-match weeks in soccer players that completed more than 60 min in the game vs. those who played less.

	>60 MINUTES PLAYED							<60 MINUTES PLAYED						
	Multi-Match Week			Single-Match Week				Multi-Match Week			Single-Match Week			
		95% CI			95% CI				95% CI			95% CI		
	Mean [SD]	Lower	Upper	Mean [SD]	Lower	Upper	*p* ^†^	Mean [SD]	Lower	Upper	Mean [SD]	Lower	Upper	*p* ^†^
CK PREGAME (U/L)	324.97 [191.4]	293.98	355.97	279.9 [158.39]	253.81	305.99	0.029 *	372.51 [233.83]	309.87	435.15	357.74 [197.96]	308.89	406.58	0.713
CK POSTGAME (U/L)	704.1 [380.84]	642.48	765.72	685.65 [320.96]	628.95	742.35	0.665	384.47 [163.88]	260.040	508.902	556.66 [291.11]	465.96	647.35	0.029 *
WEIGHT (kg)	79.51 [6.57]	78.39	80.63	79.29 [6.72]	78.36	80.21	0.761	80.48 [8.49]	78.00	82.96	80.9 [8.78]	78.92	82.89	0.790
LEAN MASS (kg)	69.26 [64.47]	68.32	70.21	69.42 [65.7]	68.65	70.18	0.804	69.94 [7.01]	68.10	71.78	70.13 [7.14]	68.69	71.57	0.872
%FAT DEXA	13.4 [1.43]	13.19	13.61	13.33 [1.46]	13.16	13.50	0.612	13.17 [1.41]	12.76	13.58	13.11 [1.68]	12.79	13.44	0.835
FATIGUE ^§^	2.22 [0.8]	2.11	2.34	2.03 [0.69]	1.94	2.13	0.015 *	2.06 [0.89]	1.84	2.29	1.95 [0.73]	1.76	2.13	0.432
MUSCLE SORENESS ^§^	2.24 [0.81]	2.13	2.36	2.08 [0.7]	1.99	2.18	0.076	2.15 [0.8]	1.95	2.34	2.01 [0.58]	1.86	2.17	0.290
STRESS ^§^	2.14 [1.08]	1.98	2.31	2.03 [1.13]	1.90	2.17	0.330	2.1 [0.95]	1.86	2.35	2.03 [0.79]	1.83	2.22	0.625
SLEEP QUALITY ^§^	2.31 [0.71]	2.19	2.43	2.24 [0.77]	2.14	2.33	0.332	2.06 [0.7]	1.85	2.28	2.09 [0.77]	1.92	2.26	0.823
SLEEP HOURS	7.19 [1.07]	7.04	7.35	7.3 [0.91]	7.17	7.43	0.282	7.29 [0.92]	7.00	7.58	7.56 [1.06]	7.33	7.79	0.151
GPS (HSR) (m)	839.07 [283.06]	792.82	885.31	868.34 [399.44]	831.10	905.58	0.333	314.69 [231.08]	259.30	370.08	301.58 [202.11]	258.30	344.86	0.713
DENSITY HSR (m/min)	9.05 [3.07]	8.29	9.80	9.53 [4.49]	8.92	10.13	0.331	13.6 [9.86]	11.417	15.781	12.72 [7.39]	11.019	14.42	0.534
GPS TOTAL DISTANCE (m)	10,256.22 [1328.31]	10,083.85	10,428.58	9911.16 [1245.2]	9772.35	10,049.96	0.002 *	2937.24 [1846.97]	2480.11	3394.37	2936.72 [1708.45]	2579.54	3293.90	0.999
DTD (m/min)	109.97 [10.3]	108.17	111.77	107.5 [11.53]	106.05	108.952	0.036 *	113.91 [14.84]	109.431	118.387	113.26 [18.57]	109.76	116.76	0.823

CK, creatine kinase; kg, kilograms; GPS, global positioning system; HSR, high-speed running; DTD, density total distance; m, meters; min, minutes; SD, standard deviation; CI, confidence interval. *p* ^†^ value: based on ANOVA, pairwise comparisons between multi-match weeks and single-match weeks; ^§^ wellbeing questionnaire, scored on a five-point Likert scale (values 1–5: 1—very good and 5—very bad); * *p* value < 0.05.

**Table 3:** Comparative assessment of muscle damage, wellbeing, body composition and match performance data in multi-match vs. single-match weeks according to player position.

		>60 MINUTES PLAYED									<60 MINUTES PLAYED								
		Multi-Match Week				Single-Match Week					Multi-Match Week				Single-Match Week				
			95% CI				95% CI									95% CI			
		Mean [SD]	Lower	Upper	*p* ^†^	Mean [SD]	Lower	Upper	*p* ^†^	*p* ^‡^	Mean [SD]	Lower	Upper	*p* ^†^	Mean [SD]	Lower	Upper	*p* ^†^	*p* ^‡^
CK PREGAME (U/L)	Defender	337.21 [199.13]	293.91	380.50	0.004 *	311.48 [147.84]	275.62	347.34	0.027 *	0.369	347.73 [209.03]	224.72	470.74	0.020 *	303.46 [102.69]	190.31	416.61	0.218	0.601
	Midfielder	295.17 [185.26]	254.23	336.10		240.58 [134.97]	206.46	274.70		0.045 *	316.46 [210]	233.18	399.74		341.38 [236.81]	276.05	406.71		0.642
	Striker	438.27 [143.76]	338.00	538.55		372.68 [299.01]	272.41	472.96		0.364	534.3 [260.48]	405.29	663.31		418.82 [149.97]	331.84	505.80		0.144
CK POSTGAME (U/L)	Defender	758.57 [441.35]	666.34	850.80	0.005 *	730.57 [342.7]	647.29	813.85	0.046 *	0.658	456 [167.04]	213.24	698.76	0.114	301.33 [135.15]	58.57	544.09	0.000 *	0.369
	Midfielder	622.19 [263.61]	529.96	714.42		600.3 [243.2]	515.71	684.90		0.731	297.4 [134.05]	164.43	430.37		464.63 [264.1]	368.17	561.10		0.046 *
	Striker	849.6 [495.83]	637.27	1061.93		958.78 [442.41]	734.96	1182.59		0.486	548.5 [66.74]	338.26	758.74		808.1 [196.17]	675.13	941.07		0.041 *
WEIGHT (kg)	Defender	81.07 [5.89]	79.74	82.40	<0.001 *	81.12 [5.76]	80.05	82.19	<0.001 *	0.956	83.01 [6.42]	79.95	86.07	<0.001 *	83.29 [7.06]	80.34	86.24	<0.001 *	0.897
	Midfielder	76.5 [5.13]	75.24	77.76		75.93 [4.99]	74.88	76.97		0.469	73.83 [4.89]	71.48	76.18		74.2 [4.16]	72.36	76.04		0.808
	Striker	88.13 [6.72]	85.21	91.05		89.76 [7.21]	87.10	92.41		0.657	89.19 [5.41]	86.13	92.25		89.22 [6.55]	87.02	91.43		0.986
LEAN MASS (kg)	Defender	70.12 [52.99]	69.04	71.20	<0.001 *	70.52 [53.17]	69.66	71.38	<0.001 *	0.570	72.19 [56.08]	69.65	74.74	<0.001 *	71.26 [58.72]	69.07	73.44	<0.001 *	0.583
	Midfielder	65.91 [44.29]	64.87	66.94		65.86 [43.45]	65.03	66.69		0.951	64.87 [42.97]	63.07	66.67		64.72 [42.89]	63.27	66.17		0.894
	Striker	78.97 [58.2]	76.95	80.99		80.51 [52.96]	78.77	82.24		0.258	76.84 [48.2]	74.46	79.22		76.54 [48.88]	74.88	78.20		0.837
%FAT DEXA	Defender	13.38 [1.36]	13.07	13.69	0.538	13.04 [1.4]	12.79	13.29	<0.001 *	0.091 *	13.1 [1.48]	12.26	13.94	0.830	13.48 [1.15]	12.75	14.20	0.436	0.091
	Midfielder	13.34 [1.5]	13.04	13.64		13.42 [1.47]	13.18	13.66		0.691	13.09 [1.74]	12.49	13.68		13.13 [2.14]	12.65	13.61		0.691
	Striker	13.71 [1.42]	13.12	14.29		14.16 [1.3]	13.65	14.66		0.253	13.38 [0.47]	12.59	14.17		12.88 [1.18]	12.33	13.43		0.253
FATIGUE ^§^	Defender	2.13 [0.61]	1.96	2.31	0.332 *	2.11 [0.59]	1.97	2.26	0.327	0.888	2.23 [0.83]	1.80	2.66	0.440	1.71 [0.46]	1.30	2.12	0.015 *	0.086
	Midfielder	2.29 [0.95]	2.13	2.46		1.96 [0.76]	1.82	2.10		0.003 *	1.91 [0.97]	1.58	2.24		1.78 [0.72]	1.52	2.03		0.533
	Striker	2.36 [0.74]	1.97	2.75		2.06 [0.74]	1.71	2.41		0.264	2.15 [0.80]	1.73	2.58		2.32 [0.74]	2.01	2.63		0.532
MUSCLE SORENESS ^§^	Defender	2.17 [0.76]	2.00	2.35	0.501 *	2.17 [0.64]	2.03	2.32	0.284	0.983	2 [0.70]	1.64	2.36	0.495	1.71 [0.46]	1.36	2.06	0.029 *	0.264
	Midfielder	2.32 [0.88]	2.15	2.49		2.01 [0.74]	1.87	2.15		0.006 *	2.14 [0.94]	1.86	2.42		1.94 [0.58]	1.73	2.16		0.286
	Striker	2.21 [0.69]	1.82	2.61		2.06 [0.74]	1.70	2.42		0.567	2.31 [0.63]	1.94	2.67		2.28 [0.54]	2.02	2.54		0.903
STRESS ^§^	Defender	2.41 [1.12]	2.15	2.66	0.014 *	2.32 [1.221]	2.11	2.53	0.001 *	0.628	1.92 [1.03]	1.46	2.39	0.347	2 [0.78]	1.55	2.45	0.090 *	0.813
	Midfielder	1.88 [1.05]	1.63	2.13		1.76 [1.04]	1.56	1.97		0.477	2.05 [1.04]	1.69	2.40		1.83 [0.84]	1.55	2.11		0.355
	Striker	2.29 [0.72]	1.71	2.86		2.06 [0.74]	1.54	2.58		0.565	2.38 [0.65]	1.92	2.85		2.32 [0.62]	1.99	2.65		0.823
SLEEP QUALITY ^§^	Defender	2.28 [0.56]	2.10	2.45	0.785 *	2.32 [0.68]	2.18	2.47	0.118	0.677	2.15 [0.68]	1.75	2.56	0.397	1.86 [0.36]	1.47	2.24	0.036 *	0.294
	Midfielder	2.36 [0.81]	2.19	2.53		2.14 [0.82]	2.00	2.28		0.047 *	1.91 [0.68]	1.60	2.22		1.97 [0.87]	1.73	2.21		0.750
	Striker	2.29 [0.82]	1.89	2.68		2.41 [0.87]	2.05	2.77		0.642	2.23 [0.72]	1.83	2.63		2.4 [0.70]	2.11	2.69		0.500
SLEEP HOURS	Defender	7.43 [0.75]	7.21	7.66	0.016 *	7.36 [0.81]	7.18	7.55	0.030 *	0.627	7.31 [0.75]	6.78	7.84	0.234	8.07 [0.82]	7.56	8.58	0.003 *	0.042 *
	Midfielder	7.05 [1.24]	6.83	7.27		7.35 [0.93]	7.16	7.53		0.045 *	7.5 [0.67]	7.09	7.91		7.72 [1.00]	7.41	8.04		0.395
	Striker	6.79 [1.25]	6.28	7.29		6.71 [1.21]	6.24	7.17		0.819	6.92 [1.32]	6.39	7.45		7.04 [1.06]	6.66	7.42		0.723
GPS (HSR) (m)	Defender	729.76 [261.54]	655.17	804.36	<0.001 *	743.78 [372.68]	684.34	803.22	<0.001 *	0.773	199.77 [180.91]	91.06	308.47	0.003 *	277.11 [174.58]	183.80	370.42	0.214	0.288
	Midfielder	941.65 [280.86]	870.12	1013.18		1006.62 [397.68]	949.41	1063.83		0.164	411.68 [239.08]	334.81	488.54		342.2 [225.77]	280.18	404.23		0.167
	Striker	831.24 [221.88]	692.12	970.36		768 [316.79]	648.17	887.83		0.499	245.52 [191.49]	143.84	347.20		262.74 [178.39]	191.94	333.54		0.784
DENSITY HSR (m/min)	Defender	8.51 [6.11]	4.18	12.84	0.013 *	12.77 [11.84]	9.05	16.48	0.239 *	0.143	7.6 [2.71]	6.78	8.42	<0.001 *	7.91 [4.24]	7.26	8.56	<0.001 *	0.562
	Midfielder	16.5 [10.55]	13.44	19.56		14.11 [6.56]	11.64	16.58		0.232	10.48 [2.96]	9.70	11.26		11.25 [4.29]	10.62	11.88		0.132
	Striker	12.96 [9.81]	8.91	17.01		10.88 [4.37]	8.06	13.70		0.407	8.67 [2.17]	7.15	10.19		8.55 [3.67]	7.24	9.87		0.910
GPS TOTAL DISTANCE (m)	Defender	10,102.58 [974.54]	9834.37	10,370.79	<0.001 *	9828.42 [723.99]	9614.71	10,042.14	<0.001 *	0.117	2686.68 [1776.43]	1780.47	3592.89	0.016 *	3391.99 [1952.37]	2614.11	4169.88	0.159	0.245
	Midfielder	10,592.38 [1545.06]	10,335.18	10,849.57		10,233.59 [1482.91]	10,027.88	10,439.30		0.033 *	3574.72 [2049.72]	2933.93	4215.51		3069.79 [1723.82]	2552.71	3586.87		0.228
	Striker	9519.08 [1146.78]	9018.86	10,019.29		8832.93 [1144.97]	8402.07	9263.80		0.042 *	2040.89 [995.67]	1193.20	2888.57		2501.2 [1478.93]	1910.95	3091.45		0.380
DTD (m/min)	Defender	105.22 [6.86]	103.23	107.21	<0.001 *	103.3 [7.52]	101.72	104.89	<0.001 *	0.140	105.22 [6.86]	103.23	107.21	<0.001 *	103.3 [7.52]	101.72	104.89	<0.001 *	0.140
	Midfielder	117.17 [8.83]	115.26	119.08		113.75 [11.95]	112.23	115.28		0.006	117.17 [8.83]	115.26	119.08		113.75 [11.95]	112.23	115.28		0.006 *
	Striker	99.24 [5.47]	95.53	102.95		97.11 [7.35]	93.92	100.31		0.393	99.24 [5.47]	95.53	102.95		97.11 [7.35]	93.92	100.31		0.393

CK, creatine kinase; kg, kilograms; GPS, global positioning system; HSR, high-speed running; DTD, density total distance; m, meters; min, minutes; SD, standard deviation. *p* ^†^ value: based on ANOVA multivariate, test results of within-subject effects according to position; ^‡^ *p* value: based on ANOVA, pairwise comparisons between multi-match weeks and single-match weeks; ^§^ wellbeing questionnaire, scored on a five-point Likert scale (values 1–5: 1—very good ratings and 5—very bad)); * *p* value < 0.05.

## Data Availability

The data presented in this study are available on request from the corresponding author.
